# Developmental Changes in Desensitisation of c-Fos Expression Induced by Repeated Maternal Separation in Pre-Weaned Mice

**DOI:** 10.1111/j.1365-2826.2012.02377.x

**Published:** 2013-01-24

**Authors:** N Horii-Hayashi, T Sasagawa, W Matsunaga, Y Matsusue, C Azuma, M Nishi

**Affiliations:** * Department of Anatomy and Cell Biology, Nara Medical UniversityKashihara, Nara, Japan; † Department of Oral and Maxillofacial Surgery, Nara Medical UniversityKashihara, Nara, Japan

**Keywords:** maternal separation, stress, c-Fos, HPA axis, corticosterone, development

## Abstract

Early-life stress has long-lasting effects on neuroendocrine and behaviour in adulthood. Maternal separation (MS) is used as a model of early-life stress and daily repeated MS (RMS) for 3 h during the first two postnatal weeks is widely used in rodent studies. However, it is not fully understood whether early-life animals desensitise/habituate to repeated stress. In the present study, we investigated the effects of daily RMS for 3 h and acute/single time MS (SMS) for 3 h on the plasma corticosterone level and c-Fos expression in the brain in mice at different postnatal ages. Mice were subjected to: (i) RMS from postnatal day (PND) 1 to 14 (RMS14); (ii) RMS from PND14 to 21 (RMS21); (iii) SMS on PND14 (SMS14); and (iv) SMS on PND21 (SMS21). Plasma corticosterone and c-Fos expression were examined on the final day in each experiment. The basal corticosterone levels in RMS14 and RMS21 were equal to those in respective age-matched controls. After the final separation, the levels were significantly increased and were comparable with those after SMS14 and SMS21, respectively. Histological analysis indicated that c-Fos expression significantly increased in many brain regions, including the paraventricular nucleus, prefrontal cortex, hippocampus, and basolateral and medial amygdale in both SMS14 and SMS21 mice. However, c-Fos expression in RMS14 mice significantly increased in many regions, whereas such increases were hardly seen in RMS21 mice. These results indicate that repeated early-life stress neither increases basal corticosterone, nor decreases the magnitude of the corticosterone response during the first three postnatal weeks, although desensitisation of c-Fos expression induced by repeated stress is changed during postnatal development.

Aberrant activity of the hypothalamic-pituitary-adrenal (HPA) axis and corticosteroid (cortisol in humans and corticosterone in rodents) release induced by adverse experiences in early life are considered to be major risk factors for the development of psychiatric disorders 1–4. Maternal separation (MS) is widely used as a laboratory model to study the mechanism underlying the relationship between early-life experiences and the development of such disorders. Cumulative evidence indicates that disruption of mother–infant interactions by MS leads to long-term effects on neuroendocrine and behaviours, which involves an enhanced stress response; increased levels of anxiety, helplessness and anhedonia; and an increased propensity for the intake of addictive drugs 5–11.

The stress response in neonatal animals differs from that in adults. The main feature in neonatal animals is the stress-hypo-responsive period (SHRP) 12–14. This period lasts approximately from postnatal day (PND) 4 to 14 in rats and from PND 1 to 12 in mice, and is characterised by a very low basal plasma corticosterone concentration and an inability to demonstrate enhanced adrenocorticotrophic hormone and corticosterone release after exposure to mild stressors 14. Disruption of mother–infant interactions by MS disinhibits the stress hypo-responsiveness 15–17. A single-time episode of MS (SMS) for 24 h in the SHRP, which is typically referred to as ‘maternal deprivation’, induces corticosterone release after exposure to subsequent mild stressors such as novelty and saline injection 15,18–20.

In addition to the long-term effects of MS on the neuroendocrine system and behaviour, several studies have demonstrated the acute effects of MS. A time-course study using 8 h of SMS at hourly intervals in PND5 mice showed that plasma corticosterone is slightly but significantly increased after 6 h of separation and that marked increases occur after separations of 7 and 8 h 21. In PND9 mice, a time-course analysis during 24 h of SMS indicated an initial significant increase of corticosterone after 4 h of separation, followed by a further gradual increase until corticosterone levels reached a maximum after 24 h 22. Similarly, corticosterone levels in PND12, 16 and 20 rats during SMS gradually increase and reach a maximum after 24 h 15. In addition to the corticosterone responses, a few studies have shown that the expression of c-Fos, an immediate early gene product and a marker of activated neurones, is induced by SMS. *In situ* hybridisation revealed that *c-fos* mRNA in the paraventricular nucleus (PVN), cingulate cortex (Cg) and piriform cortex increases after 24 h of SMS on PND 12 in rats, which indicates that some populations of neurones are activated by MS 23.

Several studies have also examined the effects of repeated MS (RMS). In many cases, RMS involves subjecting newborn rodents to daily separation for 3 h during the first two postnatal weeks 4,10,11,24,25. In adult animals, a repeated homotypic stressor generally produces desensitisation or habituation, which involves a progressive diminution of behavioural and physiological responses and is considered to be a form of non-associative learning 26. The corticosterone response induced by restraint stress in adult animals is decreased with a repetition of the same stressor 26–29 and c-Fos levels in the PVN, hippocampus, amygdala and brain stem are not increased after repetition 27,30–32. However, it is unclear whether newborn animals become desensitised to a repeated homotypic stimulus. One study showed that daily RMS for 15 min from PND1 to 14 in mice did not decrease the corticosterone response after the final separation compared to mice subjected to an initial separation on PND14 33, with the conclusion that mouse pups are not desensitised to RMS. By contrast, it has been shown that mouse pups subjected to daily RMS for 8 h from PND3 no longer show a corticosterone response or increased c-Fos expression in the PVN by PND5, whereas these changes occur after the first separation on PND5 21,34 indicating that mouse pups are rapidly desensitised to RMS.

In the present study, we examined the corticosterone response and c-Fos expression induced by RMS and SMS in pre-weaned mice, with the aim of determining whether newborn animals become desensitised to repeated maternal absence. Information on neuronal activity patterns induced by MS may also be useful for clarifying the mechanism underlying the onset of psychiatric disorders related to early-life stress in later life. Accordingly, we performed RMS and SMS with different time periods. Mice were subjected to daily RMS for 3 h from PND1 to 14 or PND14 to 21 and to SMS on PND14 or PND21 (Fig. 1a). Corticosterone levels before and after the final separation were measured by an enzyme-linked immunosorbent assay (ELISA). c-Fos expression patterns in the hypothalamus and limbic forebrain after the final separation were determined by immunohistochemistry.

**Figure 1:**
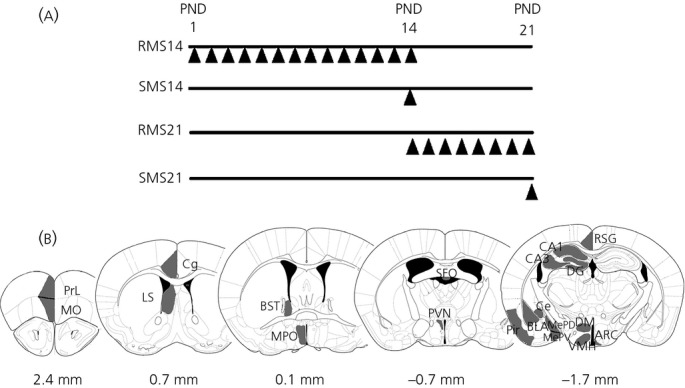
Graphical representation of maternal separation (MS) procedures and brain regions analysed for c-Fos expression. (a) Repeated MS (RMS) was performed from postnatal day (PND) 1 to 14 (RMS14) or PND14 to 21 (RMS21). Acute/single time MS (SMS) was performed on PND14 (SMS14) or PND21 (SMS21). A triangle (▴) indicates a single trial of MS. (b) Shaded regions were subjected to c-Fos expression analysis. Values under the schematic diagrams indicate the distance from the bregma line. For abbreviations, see Table 1.

## Materials and methods

### Animals

C57BL/6 female mice at day 13 of pregnancy were purchased from Japan SLC Inc. (Hamamatsu, Japan). They were individually housed and maintained under a 12 : 12 h light/dark cycle (lights on 08.00 h) at 23 °C and 55% relative humidity, with food and water available *ad lib*). The day of the pups' birth was designated as PND 0. All animal protocols were approved by the Animal Care Committee of Nara Medical University and were performed in accordance with the policies established in the NIH Guide for the Care and Use of Laboratory Animals.

### Maternal separation

Pups in the RMS group were subjected to daily MS for 3 h (09.30–12.30 h) from PND 1 to 14 or PND 14 to 21. Dams were first removed from their home cages and placed in identical new cages until the end of the separation period. Each pup was isolated in a separate cup on a heating pad maintained at 32 °C. At the end of the separation period, pups were returned to their home cages, followed by reunion with their dams. Pups in the SMS group were separated from the dam on PND 14 for 3 h from 09.30 to 12.30 h. Separation procedures were identical to those used for RMS. Pups in the control group were left undisturbed with the dam until weaning, except for cage cleaning once a week. All pups were weaned on PND 21 and housed in groups composed of three or four mice of the same sex.

### Corticosterone assay

Male mice of PND14 and 21 were sacrificed by decapitation and blood was collected from a trunk side into heparinised tubes. Pre-separation samples from RMS mice were collected at 09.30 h. In all other cases (control, post-separation, and SMS), blood was collected at 12.30 h. Plasma was obtained by centrifugation and stored at −80 °C until the day of assay. The concentration of plasma corticosterone was measured using an ELISA kit purchased from Yanaihara Inc. (Hamamatsu, Japan).

### Immunohistochemistry

Immunohistochemical methods were performed as described previously 35,36. Briefly, male mice were deeply anaesthetised with pentobarbital and then transcardially perfused with phosphate-buffered saline (PBS) followed by 4% paraformaldehyde in sodium phosphate buffer (pH 7.4). Brains were post-fixed overnight and sections (50 μm/slice) were made using a liner slicer (Pro. 7; DKS, Kyoto, Japan). After pretreatment with 0.25 mm glycine in PBS and blocking with 5% normal horse serum, sections were incubated with anti-c-Fos antibody (dilution 1 : 20000; Calbiochem, San Diego, CA, USA) for 48 h at 4 °C, followed by incubation with biotinylated anti-rabbit antibody (Vector Laboratories, Burlingame, CA, USA) for 2 h. After inactivation of endogenous peroxidase with H_2_O_2_, sections were developed using a Vectastain ABC kit (Vector Laboratories). Sections were dehydrated and coverslipped with Enthelan (Merck, Darmstadt, Germany). Observation was performed using a BX-43 trans-illuminating microscope with a FX630 CCD camera (Olympus, Tokyo, Japan).

### Quantification

Histological identification of neuronal nuclei was performed based on the mouse brain atlas. The analysed brain regions are shown schematically in Fig. 1(b). The number of c-Fos-positive cells in each region was counted using two or three sections from each animal and the results were expressed as a unilateral mean per section calculated from multiple animals. Observations were carried out with the BX-43 microscope and FX-630 CCD camera and counting was performed using the software provided with the camera.

### Statistical analysis

Analyses of corticosterone levels and c-Fos-expression was performed by two-way anova followed by Tukey multiple comparison test using JMP8 (SAS Institute Japan, Tokyo, Japan). Values are expressed as the mean ± SEM. P < 0.05 was considered statistically significant.

## Results

Time schedules of the MS interventions are shown in Fig. 1(a). Mice were subjected to (i) RMS from PND1 to 14 (RMS14); (ii) SMS on PND14 (SMS14); (iii) RMS from PND14 to 21 (RMS21); and (iv) SMS on PND 21 (SMS21). Plasma corticosterone levels and c-Fos expression in the brain were examined in these mice. Analysed brain regions are schematically represented in Fig. 1(b) and abbreviated names are listed in Table 1.

**Table 1:** Abbreviated Names of Brain Regions

ARC	Arcuate nucleus
BLA	Basolateral region of the amygdala
BST	Bed nucleus of the stria terminalis
CA1	Hippocampal CA1
CA3	Hippocampal CA3
Ce	Central amygdaloid nucleus
Cg	Cingulate cortex
DG	Dentate gyrus
DM	Dorsomedial hypothalamic nucleus
LS	Lateral septum
MePD	Posterodorsal part of the medial amygdaloid nucleus
MePV	Posteroventral part of the medial amygdaloid nucleus
MO	Medial orbital cortex
MPO	Medial preoptic area
Pir	Piriform cortex
PrL	Prelimbic cortex
PVN	Paraventricular nucleus
RSG	Retrosplenial granular cortex
SFO	Subfornical organ
VMH	Ventromedial hypothalamic nucleus

### Corticosterone

**Figure 2:**
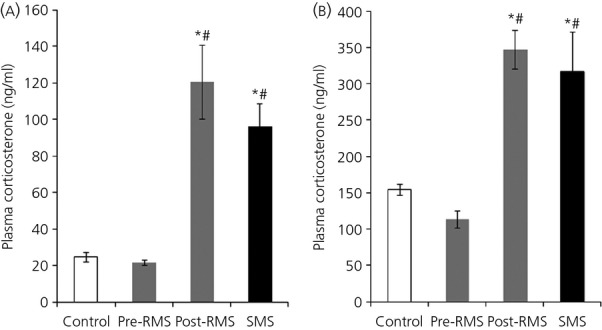
Plasma corticosterone levels of repeated MS (RMS) and acute/single time MS (SMS) mice on postnatal day (PND) 14 and PND21. The graphs show plasma corticosterone concentrations on PND14 (a) and PND21 (b) (n = 5-9 for each group). Blood samples were collected before (pre-RMS) and after (post-RMS) the final separation for RMS mice and after separation for SMS mice. *P < 0.05 versus control; ^#^P < 0.05 versus Pre-RMS. For abbreviations, see Table 1.

Corticosterone levels are shown in Fig. 2. There were significant effects of postnatal treatment in PND14 (F_3,28_ = 26.7, P < 0.0001) and PND21 (F_3,23_ = 14.9, P < 0.0001) mice. Significant increases of corticosterone were seen in post-separated RMS14 (120.6 ± 20.3 ng/ml, P = 0.02, post-hoc), SMS14 (96.2 ± 12.7 ng/ml, P = 0.02, post-hoc) (Fig. 2a), post-separated RMS21 (347.5 ± 26.7 ng/ml, P < 0.0001, post-hoc) and SMS21 (317.4 ± 54.3 ng/ml, P < 0.0001, post-hoc) mice (Fig. 2b) compared to age-matched controls (PND14: 24.9 ± 2.7 ng/ml, PND21: 154.6 ± 7.4 ng/ml). However, corticosterone levels in pre-separated RMS14 (22.0 ± 1.4 ng/ml, P = 1.0, post-hoc) and pre-separated RMS21 (113.4 ± 11.8 ng/ml, P = 0.8, post-hoc) mice, which were considered to be basal levels in RMS animals, were comparable to age-matched controls (Fig. 2).

### c-Fos expression

**Figure 3:**
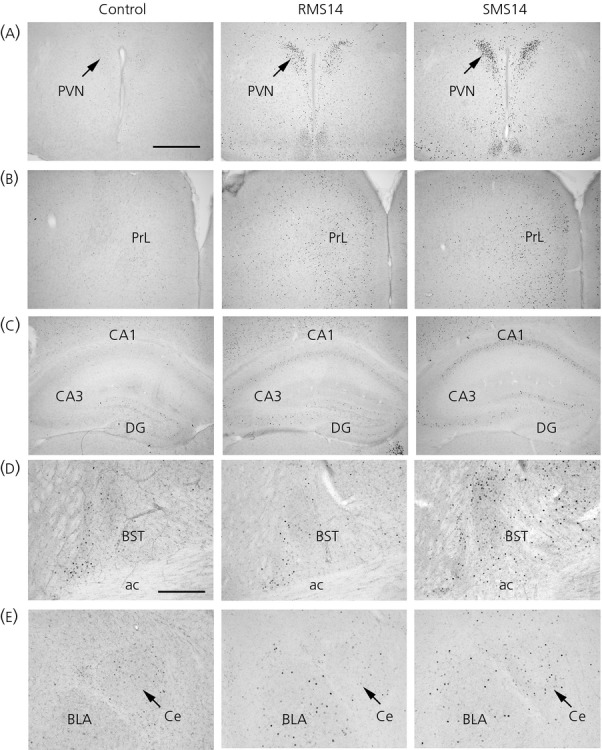
Immunohistochemical images of c-Fos expression after maternal separation (MS) on postnatal day 14. (a–e) Representative immunohistochemical images of c-Fos expression in nonseparated control (left), repeated MS (RMS) 14 (centre) and acute/single time MS (SMS) 14 (right) mice in the paraventricular nucleus (PVN) (a), prelimbic cortex (PrL) (b), hippocampal CA1 (CA1), hippocampal CA3 (CA3) and dentate gyrus (DG) in the hippocampus (c), bed nucleus of stria terminalis (BST) (d), and basolatera region of the amygdala (BLA) and central amygdaloid nucleus (Ce) in the amygdala (e). ac, anterior commissure. Scale bars = 500 (a–c), 250 μm (d, e). For abbreviations, see Table 1.

**Figure 4:**
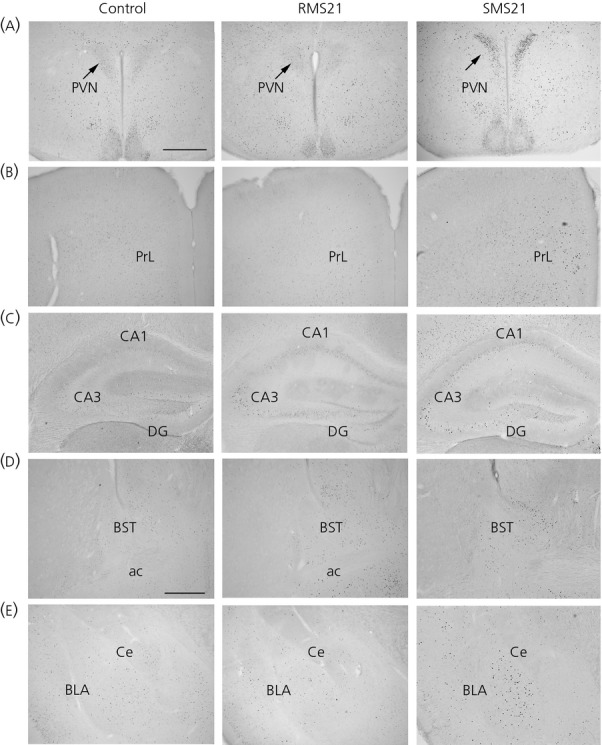
Immunohistochemical images of c-Fos expression after maternal separation (MS) on postnatal day 21. (a–e) Representative immunohistochemical images of c-Fos expression in nonseparated control (left), repeated MS (RMS) 21 (centre) and acute/single time MS (SMS) 21 (right) mice in the paraventricular nucleus (PVN) (a), prelimbic cortex (PrL) (b), hippocampal CA1 (CA1), hippocampal CA3 (CA3) and dentate gyrus (DG) in the hippocampus (c), bed nucleus of stria terminalis (BST)(d), and basolatera region of the amygdala (BLA)and central amygdaloid nucleus (Ce) in the amygdala (e) . ac, anterior commissure. Scale bars = 500 (a–c, e), 250 μm (d). For abbreviations, see Table 1.

Representative images of c-Fos expression in the brain after RMS and SMS and in controls are shown in Fig. 3 (PND14) and Fig. 4 (PND21). In RMS14 and SMS14 mice, c-Fos expression clearly increased in the PVN (Fig. 3a), prelimbic cortex (PrL) (Fig. 3b), hippocampal CA1 (CA1) and hippocampal CA3 (CA3) (Fig. 3c), and basolatera region of the amygdala (BLA) (Fig. 3e) compared to controls (Fig. 3a–c,e). c-Fos expression levels in the bed nucleus of stria terminalis (BST) (Fig. 3d) and central amygdaloid nucleus (Ce) (Fig. 3e) of SMS14 mice also increased, although those for RMS14 mice were comparable to controls (Fig. 3d,e). c-Fos levels in SMS21 mice clearly increased in the PVN (Fig. 4a), PrL (Fig. 4b), CA1 and CA3 (Fig. 4c), BST (Fig. 4d) and BLA (Fig. 4e), whereas those in the dentate gyrus (DG) (Fig. 4c) and Ce (Fig. 4e) were comparable to controls (Fig. 4a–e). c-Fos levels in RMS21 mice showed little increase in the PVN (Fig. 4a), PrL (Fig. 4b), DG (Fig. 4c), BST (Fig. 4d), BLA and Ce (Fig. 4e), a slight increase in the CA1 (Fig. 4c), and a clear increase in the CA3 (Fig. 4c) compared to controls (Fig. 4a–e).

**Figure 5:**
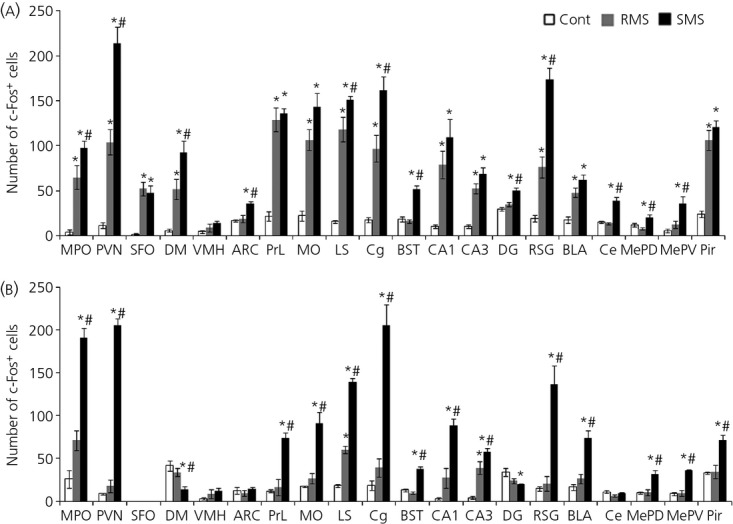
c-Fos expression in the hypothalamus and limbic forebrain after maternal separation (MS). The graphs show the numbers of c-Fos-positive cells on postnatal day (PND)14 (a) and PND21 (b) in nonseparated control (white bar), repeated MS (RMS) (grey bar) and acute/single time MS (SMS) (black bar) mice (n = 4-5 for each group). *P < 0.05 versus control; ^#^P < 0.05 versus RMS.

The results of anova and post-hoc analysis in c-Fos expression after MS are shown in Table 2 and the numbers of c-Fos-positive cells in each brain region are indicated in Fig. 5. The results of the anova showed that significant differences were found in all analysed regions except for the ventromedial hypothalamic nucleus (VMH) on PND14 and except for the subfornical organ (SFO), VMH, arcuate nucleus (ARC) and Ce on PND 21 (Table 2). Post-hoc analyses indicated that, in RMS14 mice, significant increases in c-Fos expression were found in the all region excluding the VMH, ARC, BST, DG, Ce, posterodorsal part of the medial amygdaloid nucleus (MePD) and posteroventral part of the medial amygdaloid nucleus (MePV) and, in SMS14, they were observed in the all region excluding the VMH (Fig. 5a). By contrast, in RMS21 mice, significant increases were observed only in the lateral septum (LS) and CA3, even though those in SMS21 mice were found in all regions, excluding the SFO, DM, VMH, ARC, DG and Ce.

**Table 2:** Results of anova and Post-hoc Analysis of c-Fos Expression After Maternal Separation

Region	anova			Post-hoc test					
P14	P21	Pair	P14	P21
MPO	F_2,11_ = 49.0	F_2,13_ = 41.1	Control × RMS	0.007*	0.16
< 0.0001*	< 0.0001*	Control × SMS	< 0.0001*	< 0.0001*
		RMS × SMS	0.0007*	0.0002*
PVN	F_2,11_ = 58.1	F_2,13_ = 361	Control × RMS	0.002*	0.58
< 0.0001*	< 0.0001*	Control × SMS	< 0.0001*	< 0.0001*
		RMS × SMS	0.0007*	< 0.0001*
SFO	F_2,11_ = 20.4	^a^	Control × RMS	0.0007*	^a^
0.0004*	^a^	Control × SMS	0.0013*	^a^
		RMS × SMS	0.86	^a^
DM	F_2,11_=18.2	F_2,13_ = 9.26	Control × RMS	0.02*	0.56
0.0007*	0.004*	Control × SMS	0.0005*	0.004*
		RMS × SMS	0.04*	0.03*
VMH	F_2,11_ = 2.85	F_2,13_ = 1.85	Control × RMS	^b^	^b^
0.1	0.2	Control × SMS	^b^	^b^
		RMS × SMS	^b^	^b^
ARC	F_2,11_ = 12.2	F_2,13_ = 0.56	Control × RMS	0.87	^b^
0.002*	0.58	Control × SMS	0.003*	^b^
			RMS × SMS	0.007*	^b^
PrL	F_2,11_ = 41.7	F_2,13_ = 97.7	Control × RMS	< 0.0001*	0.89
< 0.0001*	< 0.0001*	Control × SMS	< 0.0001*	< 0.0001*
		RMS × SMS	0.97	< 0.0001*
MO	F_2,11_ = 23.5	F_2,13_ = 75.3	Control × RMS	0.0006*	0.65
0.0003*	< 0.0001*	Control × SMS	0.0005*	< 0.0001*
		RMS × SMS	0.99	< 0.0001*
LS	F_2,11_ = 73.7	F_2,13_ = 325	Control × RMS	< 0.0001*	< 0.0001*
< 0.0001*	< 0.0001*	Control × SMS	< 0.0001*	< 0.0001*
		RMS × SMS	0.04*	< 0.0001*
Cg	F_2,11_ = 33.3	F_2,13_ = 49.0	Control × RMS	0.003*	0.64
< 0.0001*	< 0.0001*	Control × SMS	< 0.0001*	< 0.0001*
		RMS × SMS	0.01*	< 0.0001*
BST	F_2,11_ = 38.0	F_2,13_ = 73.5	Control × RMS	0.84	0.46
< 0.0001*	< 0.0001*	Control × SMS	0.0001*	< 0.0001*
		RMS × SMS	< 0.0001*	< 0.0001*
CA1	F_2,11_ = 11.9	F_2,13_ = 38.0	Control × RMS	0.02*	0.1
0.002*	< 0.0001*	Control × SMS	0.002*	< 0.0001*
		RMS × SMS	0.35	0.0003*
CA3	F_2,11_ = 29.3	F_2,13_ = 35.5	Control × RMS	0.0012*	0.0009*
0.0001*	< 0.0001*	Control × SMS	0.0001*	< 0.0001*
		RMS × SMS	0.15	0.46
DG	F_2,11_ = 16.3	F_2,13_ = 5.0	Control × RMS	0.41	0.13
0.001*	0.02*	Control × SMS	0.001*	0.02*
		RMS × SMS	0.006*	0.67
RSG	F_2,11_ = 60.1	F_2,13_ = 26.0	Control × RMS	0.007*	0.95
< 0.0001*	< 0.0001*	Control × SMS	< 0.0001*	0.0001*
		RMS × SMS	0.0002*	0.0003*
BLA	F_2,11_ = 22.5	F_2,13_ = 31.6	Control × RMS	0.003*	0.46
0.0003*	< 0.0001*	Control × SMS	0.0003*	< 0.0001*
		RMS × SMS	0.14	0.0003*
Ce	F_2,11_ = 30.3	F_2,13_ = 3.14	Control × RMS	0.88	^b^
< 0.0001*	0.08	Control × SMS	0.0003*	^b^
		RMS × SMS	0.0002*	^b^
MePD	F_2,11_ = 4.1	F_2,13_ = 15.6	Control × RMS	0.29	0.99
0.04*	0.0006*	Control × SMS	0.04*	0.0011*
		RMS × SMS	0.04*	0.002*
MePV	F_2,11_ = 7.8	F_2,13_ = 46.2	Control × RMS	0.66	0.97
0.01*	< 0.0001*	Control × SMS	0.01*	< 0.0001*
		RMS × SMS	0.04*	< 0.0001*
Pir	F_2,11_ = 35.4	F_2,13_ = 14.3	Control × RMS	< 0.0001*	0.98
< 0.0001*	0.0009*	Control × SMS	0.0002*	0.0015*
		RMS × SMS	0.84	0.0029*

* P < 0.05, ^a^c-Fos expression was not detected, ^b^Post-hoc analysis was not performed as a result of nonsignificant differences by anova. RMS, repeat maternal seperation; SMS, acute/single time maternal seperation. For all other abbreviations, see Table 1.

## Discussion

The results obtained in the present study show that plasma corticosterone levels in RMS14 and RMS21 mice after the final separation were equal to those in SMS14 and SMS21 mice, respectively. In addition, the pre-separated corticosterone levels (basal levels) in RMS14 and RMS21 mice were equal to the levels of age-matched controls. These results indicate that RMS neither decreases the magnitude of the corticosterone response, nor increases basal corticosterone secretion at both ages. Similar results have been found in previous studies: daily RMS for 15 min from PND1 to 14 did not decrease the corticosterone response after the final separation 33 and the basal corticosterone levels on PND3, 6, 9 and 12 were not changed by daily RMS for 3 h from birth 37. In adult animals, repetition of a homotypic stressor such as daily restraint stress for 30 min is known to cause increased basal corticosterone secretion and a decreased corticosterone response compared to acutely stressed animals 38. Therefore, the manner of corticosterone response to repeated stress is assumed to differ between early life and adulthood.

c-Fos expression analysis revealed that many brain regions were activated by MS and that the manner of c-Fos expression changed developmentally. Many regions of the hypothalamus and limbic forebrain were activated by SMS at both ages, although the manner of c-Fos expression in RMS groups differed markedly on PND14 and PND21: in RMS14 mice, the c-Fos levels in many regions were markedly increased compared to age-matched controls, except for the VMH, ARC, BST, DG, Ce, MePV and MePD; whereas, in RMS21 mice, c-Fos was suppressed to control levels in all observed brain regions, except for the LS and CA3. These results suggest that repetition of a homotypic stimulus suppresses c-Fos expression by PND21, although such suppression hardly occurs on PND14. Furthermore, in animals subjected to repeted homotypic stress during postnatal periods, enhanced adrenal secretion of corticosterone is not always correlated with increased c-fos expression in the PVN. Such a developmental difference in c-Fos expression in RMS groups may be related to a critical window in the development of stress responses, including the HPA axis, during which animals are more susceptible to MS and other environmental influences. In mice and rats, the critical window is the first 2 postnatal weeks. Therefore, a lack of repeated stress-induced suppression of c-Fos expression in early-life animals may lead to robust changes in the nature of neurones through the expression of c-Fos target genes.

The developmental difference in suppression of c-Fos expression in RMS groups may be a result of glucocorticoid effects because corticosterone analysis showed higher corticosterone levels on PND21 than those on PND14 in all groups, including the control, which is also reported in previous studies 39,40, and it is also suggests that *c-fos* gene transcription is inhibited by the complex of glucocorticoid and glucocorticoid receptor (GR) 41. This hypothesis is also related to the present evidence showing that the degree of suppression of c-Fos expression in RMS21 mice was correlated with regional expression levels of GR. The degree of suppression was most striking in the PVN, PrL, Cg and retrosplenial granular cortex (RSG), in which GR expression levels are known to be high 42,43, whereas it was not remarkable in the LS and CA3, where levels are low 42,43. However, a previous study using adrenalectomised rats suggests that glucocorticoid is not a critical regulator for repeated stress-induced suppression of c-Fos expression 28. Thus, there remains the other important possibility that suppression of c-Fos expression in RMS21 mice reflects some central aspects of stress response at the neurocircuit level rather than an intrinsic cellular down-regulation of c-Fos expression as a result of elevated corticosterone.

It is also noteworthy that suppression of increased c-Fos expression in RMS14 mice was observed in specific regions (BST, Ce, MePD and MePV). These regions form anatomical neural connections and are referred to as the extended amygdala, a region closely associated with anxiety, fear and psychiatric disorders 44. Thus, neural activity in the circuit of the extended amygdala may be suppressed by repetition of a homotypic stress even in PND14. Moreover, in the SFO, in which neurones are influenced by osmolarity, calcium and sodium concentrations in the systemic circulation 45, increased c-Fos expression was observed in both RMS14 and SMS14 mice compared to controls on PND14, although there were no changes in any groups on PND21. This difference may reflect increased resistance with physical growth to hyperosmolarity caused by a lack of lactation. A similar expression pattern was also observed in the DM, which is related to feeding, drinking and body weight regulation 46. The age-related changes were also seen in the Ce, which controls various fear responses, including behaviour and autonomic and endocrine regulation 47, and increased c-Fos expression was observed in SMS14 mice, whereas there was no change in SMS21 mice.

In previous studies, c-Fos expression and a corticosterone response were no longer observed on PND5 when rat pups were subjected to daily RMS for 8 h from PND3, suggesting that newborn rodents are rapidly desensitised to maternal absence 21,34. We did not examine the corticosterone level and c-Fos expression on PND5, and thus it is unclear whether mice pups on PND5 are desensitised to daily RMS for 3 h. However, our data show that mice on PND14 were not desensitised to daily RMS for 3 h from birth, based on c-Fos expression and the corticosterone response. Differences in experimental conditions, such as time of separation, age at testing, frequency of repetition and separation conditions (isolation or with a littermate), may have influenced these results. Therefore, desensitisation of pre-weaned rodents to repeated maternal absence may differ depending on the experimental conditions, and further systematic studies are needed to understand desensitisation to repeated stress in early life.

## References

[b1] McEwen BS (2003). Early life influences on life-long patterns of behavior and health. Ment Retard Dev Disabil Res Rev.

[b2] Heim C, Newport DJ, Mletzko T, Miller AH, Nemeroff CB (2008). The link between childhood trauma and depression: insights from HPA axis studies in
humans. Psychoneuroendocrinology.

[b3] Lupien SJ, McEwen BS, Gunnar MR, Heim C (2009). Effects of stress throughout the lifespan on the brain, behaviour and
cognition. Nat Rev Neurosci.

[b4] Ladd CO, Huot RL, Thrivikraman KV, Nemeroff CB, Plotsky PM (2004). Long-term adaptations in glucocorticoid receptor and mineralocorticoid receptor mRNA
and negative feedback on the hypothalamo-pituitary-adrenal axis following neonatal maternal
separation. Biol Psychiatry.

[b5] Biagini G, Pich EM, Carani C, Marrama P, Agnati LF (1998). Postnatal maternal separation during the stress hyporesponsive period enhances the
adrenocortical response to novelty in adult rats by affecting feedback regulation in the CA1
hippocampal field. Int J Dev Neurosci.

[b6] Brake WG, Zhang TY, Diorio J, Meaney MJ, Gratton A (2004). Influence of early postnatal rearing conditions on mesocorticolimbic dopamine and
behavioural responses to psychostimulants and stressors in adult rats. Eur J Neurosci.

[b7] Moffett MC, Vicentic A, Kozel M, Plotsky P, Francis DD, Kuhar MJ (2007). Maternal separation alters drug intake patterns in adulthood in rats. Biochem Pharmacol.

[b8] Plotsky PM, Thrivikraman KV, Nemeroff CB, Caldji C, Sharma S, Meaney MJ (2005). Long-term consequences of neonatal rearing on central corticotropin-releasing factor
systems in adult male rat offspring. Neuropsychopharmacology.

[b9] Zhang TY, Chretien P, Meaney MJ, Gratton A (2005). Influence of naturally occurring variations in maternal care on prepulse inhibition
of acoustic startle and the medial prefrontal cortical dopamine response to stress in adult
rats. J Neurosci.

[b10] Uchida S, Hara K, Kobayashi A, Funato H, Hobara T, Otsuki K, Yamagata H, McEwen BS, Watanabe Y (2010). Early life stress enhances behavioral vulnerability to stress through the activation
of REST4-mediated gene transcription in the medial prefrontal cortex of rodents. J Neurosci.

[b11] Marais L, van Rensburg SJ, van Zyl JM, Stein DJ, Daniels WM (2008). Maternal separation of rat pups increases the risk of developing depressive-like
behavior after subsequent chronic stress by altering corticosterone and neurotrophin levels in the
hippocampus. Neurosci Res.

[b12] Levine S (1994). The ontogeny of the hypothalamic-pituitary-adrenal axis. The influence of maternal
factors. Ann NY Acad Sci.

[b13] Schapiro S, Geller E, Eiduson S (1962). Neonatal adrenal cortical response to stress and vasopressin. Proc Soc Exp Biol Med.

[b14] Schmidt MV, Enthoven L, van der Mark M, Levine S, de Kloet ER, Oitzl MS (2003). The postnatal development of the hypothalamic-pituitary-adrenal axis in the
mouse. Int J Dev Neurosci.

[b15] Stanton ME, Gutierrez YR, Levine S (1988). Maternal deprivation potentiates pituitary-adrenal stress responses in infant
rats. Behav Neurosci.

[b16] Kuhn CM, Pauk J, Schanberg SM (1990). Endocrine responses to mother-infant separation in developing rats. Dev Psychobiol.

[b17] Hofer MA (1994). Early relationships as regulators of infant physiology and behavior. Acta Paediatr Suppl.

[b18] Rosenfeld P, Gutierrez YA, Martin AM, Mallett HA, Alleva E, Levine S (1991). Maternal regulation of the adrenocortical response in preweanling
rats. Physiol Behav.

[b19] Cirulli F, Santucci D, Laviola G, Alleva E, Levine S (1994). Behavioral and hormonal responses to stress in the newborn mouse: effects of maternal
deprivation and chlordiazepoxide. Dev Psychobiol.

[b20] Levine S, Huchton DM, Wiener SG, Rosenfeld P (1991). Time course of the effect of maternal deprivation on the
hypothalamic-pituitary-adrenal axis in the infant rat. Dev Psychobiol.

[b21] Enthoven L, Oitzl MS, Koning N, van der Mark M, de Kloet ER (2008). Hypothalamic-pituitary-adrenal axis activity of newborn mice rapidly desensitizes to
repeated maternal absence but becomes highly responsive to novelty. Endocrinology.

[b22] Schmidt M, Enthoven L, van Woezik JH, Levine S, de Kloet ER, Oitzl MS (2004). The dynamics of the hypothalamic-pituitary-adrenal axis during maternal
deprivation. J Neuroendocrinol.

[b23] Smith MA, Kim SY, van Oers HJ, Levine S (1997). Maternal deprivation and stress induce immediate early genes in the infant rat
brain. Endocrinology.

[b24] Lippmann M, Bress A, Nemeroff CB, Plotsky PM, Monteggia LM (2007). Long-term behavioural and molecular alterations associated with maternal separation
in rats. Eur J Neurosci.

[b25] Chocyk A, Przyborowska A, Dudys D, Majcher I, Mackowiak M, Wedzony K (2011). The impact of maternal separation on the number of tyrosine hydroxylase-expressing
midbrain neurons during different stages of ontogenesis. Neuroscience.

[b26] Grissom N, Bhatnagar S (2009). Habituation to repeated stress: get used to it. Neurobiol Learn Mem.

[b27] Stamp JA, Herbert J (1999). Multiple immediate-early gene expression during physiological and endocrine
adaptation to repeated stress. Neuroscience.

[b28] Melia KR, Ryabinin AE, Schroeder R, Bloom FE, Wilson MC (1994). Induction and habituation of immediate early gene expression in rat brain by acute
and repeated restraint stress. J Neurosci.

[b29] Carter RN, Pinnock SB, Herbert J (2004). Does the amygdala modulate adaptation to repeated stress?. Neuroscience.

[b30] Chen X, Herbert J (1995). Regional changes in c-fos expression in the basal forebrain and brainstem during
adaptation to repeated stress: correlations with cardiovascular, hypothermic and endocrine
responses. Neuroscience.

[b31] Stamp J, Herbert J (2001). Corticosterone modulates autonomic responses and adaptation of central
immediate-early gene expression to repeated restraint stress. Neuroscience.

[b32] Umemoto S, Kawai Y, Ueyama T, Senba E (1997). Chronic glucocorticoid administration as well as repeated stress affects the
subsequent acute immobilization stress-induced expression of immediate early genes but not that of
NGFI-A. Neuroscience.

[b33] D'Amato FR, Cabib S, Puglisi-Allegra S, Patacchioli FR, Cigliana G, Maccari S, Angelucci L (1992). Effects of acute and repeated exposure to stress on the
hypothalamo-pituitary-adrenocortical activity in mice during postnatal development. Horm Behav.

[b34] Daskalakis NP, Claessens SE, Laboyrie JJ, Enthoven L, Oitzl MS, Champagne DL, de Kloet ER (2011). The newborn rat's stress system readily habituates to repeated and prolonged maternal
separation, while continuing to respond to stressors in context dependent fashion. Horm Behav.

[b35] Horii-Hayashi N, Okuda H, Tatsumi K, Ishizaka S, Yoshikawa M, Wanaka A (2008). Localization of chondroitin sulfate proteoglycan versican in adult brain with special
reference to large projection neurons. Cell Tissue Res.

[b36] Horii-Hayashi N, Tatsumi K, Matsusue Y, Okuda H, Okuda A, Hayashi M, Yano H, Tsuboi A, Nishi M, Yoshikawa M, Wanaka A (2010). Chondroitin sulfate demarcates astrocytic territories in the mammalian cerebral
cortex. Neurosci Lett.

[b37] Lajud N, Roque A, Cajero M, Gutiérrez-Ospina G, Torner L (2012). Periodic maternal separation decreases hippocampal neurogenesis without affecting
basal corticosterone during the stress hyporesponsive period, but alters HPA axis and coping
behavior in adulthood. Psychoneuroendocrinology.

[b38] Hill MN, McLaughlin RJ, Bingham B, Shrestha L, Lee TT, Gray JM, Hillard CJ, Gorzalka BB, Viau V (2010). Endogenous cannabinoid signaling is essential for stress adaptation. Proc Natl Acad Sci USA.

[b39] Henning SJ (1978). Plasma concentrations of total and free corticosterone during development in the
rat. Am J Physiol.

[b40] Moriceau S, Roth TL, Sullivan RM (2010). Rodent model of infant attachment learning and stress. Dev Psychobiol.

[b41] Herdegen T, Leah JD (1998). Inducible and constitutive transcription factors in the mammalian nervous system:
control of gene expression by Jun, Fos and Krox, and CREB/ATF proteins. Brain Res Brain Res Rev.

[b42] Morimoto M, Morita N, Ozawa H, Yokoyama K, Kawata M (1996). Distribution of glucocorticoid receptor immunoreactivity and mRNA in the rat brain:
an immunohistochemical and *in situ* hybridization study. Neurosci Res.

[b43] Ahima RS, Harlan RE (1990). Charting of type II glucocorticoid receptor-like immunoreactivity in the rat central
nervous system. Neuroscience.

[b44] Davis M, Walker DL, Miles L, Grillon C (2010). Phasic vs sustained fear in rats and humans: role of the extended amygdala in fear vs
anxiety. Neuropsychopharmacology.

[b45] Smith PM, Ferguson AV (2010). Circulating signals as critical regulators of autonomic state–central roles
for the subfornical organ. Am J Physiol Regul Integr Comp Physiol.

[b46] Bellinger LL, Bernardis LL (2002). The dorsomedial hypothalamic nucleus and its role in ingestive behavior and body
weight regulation: lessons learned from lesioning studies. Physiol Behav.

[b47] Ledoux JE, Muller J (1997). Emotional memory and psychopathology. Philos Trans R Soc Lond B Biol Sci.

